# Half-Yearly Report of Cases in Midwifery, Which Have Occurred in the Northern District of the London and Southwark Midwifery Institution

**Published:** 1832-08

**Authors:** C. Waller

**Affiliations:** Consulting Accoucheur to the above Institution, and Lecturer on Midwifery at 93, Bartholomew close.


MIDWIFERY.

*Half-yearly Report of Cases in Midwifery, which have occurred in the Northern District of the London and Southwark Midwifery Institution.* By C. WALLER, M.D., Consulting Accoucheur to the above Institution, and Lecturer on Midwifery at 93, Bartholomew close.

| 1832. | Number of Women delivered. | Sex of Children. | | Born Alive. | Stillborn. | Presentation. |
|---|---|---|---|---|---|---|
| | | Males. | Females. | | | |
| January... | 59* | 32 | 29 | 58 | 3 | 58 Natural<br>1 Foot<br>1 Face to Pubis<br>1 Arm |
| February . | 38 | 22 | 16 | 35 | 3 | 35 Natural<br>3 Breech |
| March ... | 44 | 23 | 21 | 43 | 1 | 42 Natural<br>2 Face to Pubis |
| April ...... | 44 | 24 | 20 | 42 | 2 | 43 Natural<br>1 Arm & Head |
| May .. ... | 41† | 23 | 19 | 38 | 4 | 38 Natural<br>2 Breech<br>2 Face to Pubis |
| June ...... | 44 | 28 | 16 | 43 | 1 | 43 Natural<br>1 Breech |
| Total ... | 270 | 152 | 121 | 259 | 14 | * Two cases of twins.<br>† One case of twins. |

Several of the above, reported as Stillborn, were Premature Births.

MANY cases of considerable interest have occurred during the last half-year, but want of time has again prevented their being recorded so fully as they ought to have been.

We have not been quite free from puerperal fever, five cases having occurred, three of which ended fatally. They were treated by local bleeding and the free administration of mercury: ptyalism was induced in those which terminated favorably, but not in the other, although large doses of calomel were employed, (twenty grains, with four grains of opium, repeated several times,) together with mercurial frictions.

In three instances craniotomy was performed: in the first instance, there was distortion of the pelvis to a considerable extent.

The second case was attended with some anomalous symptoms not easily accounted for. The os uteri was, at the commencement of the labour, rather rigid, though not extraordinarily so; the patient complained greatly of her pains. The liquor amnii came away in the latter part of the afternoon, and before bedtime I saw the patient: the labour was proceeding slowly, and I left a pupil in attendance. About two in the morning I was again summoned, and found that advancement to a certain extent had taken place; every thing seemed going on well, though slowly, but the pulse was gradually increasing in frequency. At four o'clock it was beating at the rate of 140 in the minute; the female was getting very irritable, and the abdomen rather tender: I therefore determined to delay no longer, notwithstanding the comparative shortness of the time she had been in labour. It appeared to me that the hurry of the circulation could only be accounted for by some injurious pressure upon the soft parts, and therefore that the use of the long forceps would be attended with risk. Under these circumstances the perforator was used. The pulse declined almost immediately after the extraction of the child, and the female recovered without an unpleasant symptom.

In the third case there was a descent of the whole arm with the head; the pains were severe, and the parts were, in consequence, jammed into the upper aperture of the pelvis. I introduced the long forceps; but, finding the head did not descend with the exertion of a moderate degree of force, I desisted, and finished the delivery by opening the head. The patient had a smart attack of fever, from which she appeared to be recovering; but on the ninth day abdominal symptoms came on, and on the eleventh she died.

An interesting case of convulsions has occurred, the patient not being seized until the fourth day after delivery. This woman complained of much pain in the uterine region on the preceding evening, which was relieved by taking three opium pills, (one grain and a half in each.) The paroxysms succeeded each other very quickly, not lasting long at a time, but she was seldom free for more than thirty seconds at a time. Bleeding from the arm, leeching the temples, and active purgation, eventually cured her, but she remained in a half-stupid state for several days. Had the opium any thing to do with the production of these convulsions?

I hope in future to be enabled to send a more detailed report of our charity: our numbers are progressively on the increase, and important cases are frequently occurring.

*Bartholomew Close; July* 1832.

